# Visceral and emotional responses to direct electrical stimulations of the cortex

**DOI:** 10.1002/acn3.51694

**Published:** 2022-11-24

**Authors:** Hugo Soulier, François Mauguière, Hélène Catenoix, Alexandra Montavont, Jean Isnard, Marc Hermier, Marc Guenot, Sylvain Rheims, Laure Mazzola

**Affiliations:** ^1^ Department of Neurology University Hospital St Etienne France; ^2^ Lyon Neurosciences Research Center (CRNL) INSERM U1028, CNRS UMR5292 and Lyon 1 University Lyon France; ^3^ Department of Functional Neurology and Epileptogy Hospices Civils de Lyon and Lyon 1 University Lyon France; ^4^ Department of Neuroradiology, East Group Hospital, Hospices Civils de Lyon Lyon France; ^5^ Department of Functional Neurosurgery Hospices Civils de Lyon and Lyon 1 University Lyon France

## Abstract

**Objective:**

Visceral sensations are bodily symptoms which are component manifestations of emotions frequently reported during epileptic seizures.

Nowadays, the underlying mechanism and location of brain areas involved in the processing of these sensations remain unclear. Our objectives were to characterize the type and frequency of visceral and emotional responses evoked by electrical stimulations, to produce a mapping of brain structures involved in their processing, and to assess the link between visceral sensations and emotional feelings.

**Methods:**

We reviewed 12,088 bipolar stimulations performed in 203 patients during the presurgical evaluation of drug refractory epilepsy. Responses to stimulation were divided into viscero‐sensitive, viscero‐vegetative, and emotional sensations. Univariate analysis and conditional logistic regression were used to assess the association between visceral and emotional sensations and localization of the stimulated contacts.

**Results:**

In total, 543 stimulations evoked visceral and emotional sensations. Stimulations of operculo‐insulolimbic structures (amygdala, anterior and posterior insula, anterior and mid‐cingulate cortex, hippocampus, parahippocampus, temporal pole, frontal and parietal operculum) were significantly more associated with visceral and emotional sensations than all other cortical regions.

Preferential implication of certain brain structures, depending on the type of visceral responses was evidenced: temporo‐mesial structures, insula, and frontoparietal operculum for viscero‐sensitive sensations; amygdala, insula, anterior and mid‐cingulate cortex, and temporal pole for viscero‐vegetative sensations; temporo‐mesial structures, anterior cingulate cortex, and frontal operculum for emotional sensations.

**Interpretation:**

Our data can help to guide SEEG explorations when visceral or emotional symptoms are part of the ictal semiology. They also bring some insights into the mechanisms of visceroception and the functional significance of the co‐localization of visceral and emotional representations in the human brain.

## Introduction

Visceral and emotional sensations (VES) have been frequently reported as ictal manifestations of epileptic seizures. Visceral sensations refer to the perception of bodily signals arising specifically from the viscera such as the heart, lungs, stomach, or bladder.^1^ They encompass a large range of different symptoms, that we divided into three groups as already proposed in literature^2^, ^3^, ^4^: (i) viscero‐sensitive sensations, including all feelings localized in throat, esophagus, thorax, or abdomen, such as sensations of heaviness, constrictions, ascending sensations or even pain and (ii) viscero‐vegetative sensations, such as flush, nausea, feeling of tachycardia, and dyspnea. (iii) The term “emotional sensations” refers to emotional responses associated or not with a visceral sensation.^5^

Visceral, viscero‐vegetative, and emotional sensations have been reported in different types of focal seizures. In mesial temporal lobe seizures, visceral sensations are frequent, especially the viscero‐sensitive abdominal sensation, known as “epigastric aura,” which is the most frequently reported ictal symptom in up to 67% of patients.^5^, ^6^, ^7^ In insular lobe seizures, visceral symptoms are a common early manifestation and especially laryngeal discomfort or constriction,^2^ suggestive of an ictal discharge located in the anterior part of insula.^4^, ^8^ On the contrary, very few viscero‐sensitive sensations are reported in frontal lobe epilepsy, although some epigastric auras have been described.^7^ Conversely viscero‐vegetative manifestations such as dyspnea, flush or the feeling of tachycardia, urinary urge are reported, mainly in basal frontal lobe seizures, and negative emotions such as fear or anxiety are frequent.^9^, ^10^, ^11^, ^12^

In literature, several studies have reported visceral or emotional sensations by studying the stimulation of a single brain area such as the insula,^13^, ^14^ the cingulate cortex,^15^ or the temporal lobe (especially the temporal pole and mesial temporal lobe).^16^, ^17^ In our study, we did not select a brain region, but we reported all the stimulations having evoked VES, after stimulations performed all over cortical structures. This is the first study to functionally map VES after stimulating the whole cortical mantle. We systematically reviewed viscero‐sensitive, viscero‐vegetative, and emotional responses evoked by 13,078 electrical stimulations performed all over the cortical mantle, in a large series of non‐selected consecutive patients with drug‐resistant focal epilepsy who underwent a stereo‐electro‐encephalographic (SEEG). The aims of our study were to characterize the type and frequency of VES triggered by stimulations, to produce a mapping of brain structures involved in their processing, and to assess their link with the occurrence of emotional feelings.

## Methods

### Patients

We performed a retrospective observational study in patients who underwent SEEG at the Department of Functional Neurology and Epileptology, Hospices Civils de Lyon (France) between September 2000 and October 2019 as part of presurgical assessment of drug‐resistant focal epilepsy and for whom complete medical files, EEG, and video data were available.

Electrical stimulations of the cortex were performed as part of the routine of SEEG procedure used to evaluate the epileptic threshold in the epileptogenic area and to map the functionality of the implanted areas. All patients were fully informed of the purpose and risks of SEEG procedure and gave their written consent. The local ethics committee of Hospices Civils de Lyon approved this study.

### Electrode implantation and stimulation sites location

The choice of the intracranial electrodes locations was decided in order to localize the epileptogenic zone and functionally eloquent areas by a multidisciplinary team involving neurologists and neurosurgeons, based on the results of presurgical investigations including: video‐EEG recordings of seizures, interictal fluorodeoxyglucose PET, interictal and ictal single photon emission computed tomography, and brain MRI data.

The stereotactic implantation followed the procedures described in previous studies.^2^, ^18^ Electrodes were implanted perpendicular to mid‐sagittal plane with additional oblique trajectories since 2015 and were left in place chronically up to 15 days. The electrodes had a diameter of 0.8 mm and contained from 5 to 15 recording contacts. Contacts were 2 mm long and separated by 1.5 mm from one another.

From 2000 to 2008, a postimplantation frontal x‐ray at scale 1 was performed and superimposed on individual T1‐weighted brain MRI to check for the final position of each electrode with respect to the targeted anatomical structures and to localize each contact. Since 2009, MRI‐compatible electrodes have been used. Postimplantation MRI was systematically performed with electrodes in situ to identify the anatomical position of contacts.

The location of each stimulation site was that of the point located halfway between the two adjacent contacts used for bipolar stimulation (i.e., the center of the sphere of neural elements activated by electrical stimulation).

### Stimulation paradigm

Electrical stimulations were delivered while patients were awake, seated in bed, and instructed to keep their eyes open and relaxed. A current‐regulated neurostimulator delivered square pulses of current between two adjacent contacts (bipolar stimulation).

Depending on the stimulated brain area and the clinical/EEG responses, electrical stimulations could be delivered by train or by shocks. Parameters of electrical stimulation were, pulses of 0.3–0.5 ms, delivered at a frequency of 50 Hz during 3 to 5 seconds for trains, and a series of 30 brief pulses (1 ms) at 1 Hz for shocks. Stimulus intensity was from 0.2 mA to a maximum of 5 mA for trains and of 6 mA for shocks.

When bipolar stimulations were performed several times on the same contact, with increasing intensity, we included only the stimulation performed at the minimal intensity necessary to evoke a clinical response (at stimulation threshold). When no clinical response was evoked, we included only the stimulation at the highest intensity performed on these contacts.

Stimulations applied within a brain lesion or evoking an after discharge were excluded from analysis. After‐discharge was defined as the persistence of rhythmic spikes or polyspikes on stimulated contacts for more than 5 seconds after the end of the stimulation and/or their spreading to other contacts than those stimulated.

### Collection and processing of data

For each patient, age, gender, and date of the SEEG were collected. Electrical stimulations were performed by clinicians at the patient's bedside during stimulation sessions, with a constant questioning of the sensations elicited by stimulations. All sessions were video‐recorded and subjective reports and clinical observations were collected immediately after each electrical stimulation and reported in patient's medical file. As this study is retrospective, no systematic questionnaire was initially performed.

When a VES was evoked, we analyzed for each stimulation, the medical file reports, the videos, and EEG raw data. The specific characteristics of the sensation, its localization, and intensity were collected.

To determine the brain structures related to the different visceral and emotional sensations, we proceeded in three steps.

The first step consisted of looking at all the stimulations evoking any kind of visceral or emotional sensations. When a given stimulation evoked more than one type of VES at a time (e.g., abdominal discomfort and fear), the two types of sensation were counted in each VES category, so that the total number of evoked sensations is greater than that of stimulations which produced a response. The second step was to look more precisely which sensations were evoked in order to divide them into three categories: viscero‐sensitive, viscero‐vegetative, and emotional responses.

In order to classify the localization of each stimulation site, 27 distinct structures have been individualized (see Table 1). Within our classification, we grouped six strongly connected structures (anterior and mid‐cingulate cortex, amygdala, hippocampus and parahippocampus, temporal pole) under the heading “limbic system.” We also grouped four structures (anterior and posterior insula, frontal operculum, and parietal operculum) under the heading “operculo‐insular regions.” The frequency of VES in the limbic system and operculo‐insular regions could thus be compared to that obtained in other structures regrouped under the term “extra operculo‐insulo‐limbic structures” (see Table 2).

**Table 1:** Distribution of visceral and emotional sensations produced by stimulation of all brain regions.

Total number of stimulations per lobe, *N* = 12 740	Total number of stimulations per lobar subdivision	Total number of stimulations per region of interest, *N* = 12 740	VES, *N* = 543	Odd Ratios
Frontal *N* = 3726	Frontal mesial *N* = 1575	**Anterior cingulate cortex** *N* = 464	*N* = 26 (5.6%)	OR = 2.7 [1.3–5.9]
		**Midcingular cortex** *N* = 74	*N* = 4 (5.4%)	OR = 7.0 [2.3–23.8]
		Internal prefrontal cortex (F1) *N* = 188	*N* = 3 (1.6%)	NS
		Internal prefrontal orbito‐frontal cortex *N* = 649	*N* = 11 (1.7%)	NS
	Frontolateral *N* = 2151	**Frontal operculum** *N* = 479	*N* = 49 (10.2%)	OR = 4.4 [2.2–8.8]
		Motor cortex *N* = 200	*N* = 2 (1%)	NS
		Lateral prefrontal orbitofrontal cortex *N* = 132	*N* = 2 (1.5%)	NS
		Lateral prefrontal ventro‐dorsal cortex *N* = 1322	*N* = 9 (0.7%)	NS
		Premotor cortex *N* = 218	*N* = 1 (0.4%)	NS
Insula *N* = 1177	Anterior insula *N* = 842	**Anterior insula** *N* = 842	*N* = 114 (13.6%)	OR = 4.7 [2.6–8.6]
	Posterior Insula *N* = 335	**Posterior Insula** *N* = 335	*N* = 40 (12%)	OR = 6.2 [3.3–12]
Parietal *N* = 948	Lateral parietal lobe *N* = 720	Post central gyrus *N* = 70	*N* = 1 (1.4%)	NS
		Inferior parietal lobule *N* = 299	*N* = 2 (0.6%)	NS
		Superior parietal lobule *N* = 141	*N* = 4 (2.8%)	NS
		**Parietal operculum** *N* = 210	*N* = 38 (18%)	OR = 4.3 [2.1–8.9]
	Mesial Parietal lobe *N* = 228	Posterior cingulate cortex *N* = 189	*N* = 14 (7.4%)	NS
		Precuneus *N* = 39	*N* = 1 (2.5%)	NS
Temporal *N* = 6237	Temporal Pole *N* = 735	**Temporal Pole** *N* = 735	*N* = 26 (3.5%)	OR = 2.5 [1.2–5.0]
	Basal Temporal lobe *N* = 948	**T3 and T4** *N* = 948	*N* = 17 (1.9%)	Reference [OR = 1]
	Lateral temporal lobe *N* = 2007	Temporal operculum *N* = 285	*N* = 9 (3.1%)	NS
		T1 *N* = 881	*N* = 9 (1.1%)	NS
		T2 *N* = 841	*N* = 10 (1.2%)	NS
	Mesial temporal lobe *N* = 2547	**Amygdala** *N* = 697	*N* = 71 (10.1%)	OR = 8.4 [4.5–15.5]
		**Hippocampus** *N* = 1230	*N* = 60 (4.8%)	OR = 4.3 [2.3–7.8]
		**Parahippocampus** *N* = 620	*N* = 20 (3.2%)	OR = 3.1 [1.5–6.3]
Occipital *N* = 652	Internal occipital lobe		No response	No response
	Lateral occipital cortex		No response	No response

The VES column represents the distribution of VES for each brain region, expressed in absolute number and in percentages of all electrical stimulations that induced VES. The OR column represents VES expressed in odd ratios. Brain regions in bold are those for which stimulation evoked significantly more VES than others. NS, Non Significant.

**Table 2:** Distribution of each type of VES within limbic system and operculo‐insular region.

		Visceral sensations (*n* = 325)	Viscero‐vegetative sensations (*n* = 103)	Emotional sensations (*n* = 115)
	Anterior cingulate cortex *N* = 464	*N* = 9 (1.9%) OR = NS	***N* = 6 (1.3%) OR = 7.7 [1,9–31]**	***N* = 16 (3.4%) OR = 9.6 [4.0–23.0]**
	Midcingulate cortex *N* = 74	*N* = 3 (4.0%) OR = NS	***N* = 1 (1.35%) OR = 13.2 [1.3–95]**	No response
Limbic system	Amygdala *N* = 697	***N* = 46 (6.5%) OR = 7 [4.7–12.9]**	***N* = 24 (3.4%) OR = 26 [11.5–61]**	***N* = 16 (2.3%) OR = 13.8 [5.6–33]**
	Hippocampus *N* = 1230	***N* = 34 (2.7%) OR = 3.8 [2.3–6.4]**	***N* = 13 (1%) OR = 5.4 [2.2–13.2]**	***N* = 16 (1.3%) OR = 4.2 [1.8–9.6]**
	Parahippocampus *N* = 620	***N* = 12 (1.9%) OR = 3.2 [1.5–6.7]**	*N* = 6 (0.9%) OR = NS	*N* = 2 (0.3%) OR = NS
	Temporal Pole *N* = 735	*N* = 6 (0.8%) OR = NS	***N* = 5 (0.7%) OR = 5.2 [1.8–15.3]**	*N* = 6 OR = NS
Operculo‐insular region	Frontal operculum *N* = 479	***N* = 33 (6.8%) OR = 5.6 [3.2–10.0]**	*N* = 2 (0.41%) OR = NS	***N* = 7 (1.4%) OR = 2.7 [1.1–7.7]**
	Parietal operculum *N* = 210	***N* = 13 (6.1%) OR = 3.5 [1.7–7.3]**	No response	No response
	Anterior insula *N* = 842	***N* = 80 (9.5%) OR = 5.6 [3.6–8.7]**	***N* = 21 (2.5%) OR = 14.2 [6.1–33.2]**	***N* = 21 (2.5%) OR = 4,2 [2.1–8.3]**
	Posterior insula *N* = 335	***N* = 27 (8.5%) OR = 5.6 (3.1–10)**	***N* = 10 (3%) OR = 12.1 (4.22–34)**	*N* = 3 (0.9%) OR = NS
Extra operculo‐insulo‐limbic	Reference (OR = 1)	OR = 1	OR = 1	OR = 1

Values are expressed in absolute number, percentages of responses, and odd ratios. NS: Nonsignificant. For each type of VES, the insulo‐limbic structures where stimulation evoked significantly VES, are in bold.

Finally, we focused on the localization of contacts in which throat constriction, ascending epigastric, and abdominal pain sensations could be obtained as they are visceral sensations frequently reported during epileptic seizures.

In order to illustrate our results, we used MRIcroGl software which allows a three‐dimensional representation of the brain and structures of interest.

### Statistical Analyses

Statistical analysis was made with SAS 9.4. The Chi‐square test was used to assess the relation between two qualitative variables and the Student's test was used to compare averages after normality test. We first assessed the association between VES and localization of the stimulated contacts with an univariate analysis. Then a multivariate analysis with a conditional logistic regression with a statistical adjustment for patient was carried out. Age, gender, lateralization of the stimulation, modality of stimulation (trains or shocks), stimulation intensity were included in this statistical model. The significant threshold was fixed at 5%.

Statistical analysis of the occurrence of VES was made for each of the brain regions listed in Table 1, and odd ratios (ORs) were calculated. To compare the different structures, one with the other, the structure with the lowest OR was chosen as the reference structure. Considering all VES, temporal inferior (T3) and fusiform (T4) gyri had the lowest OR and were so chosen as the reference (OR = 1). Other structures ORs were so calculated using this reference and ordered according to their relative OR.

Then, in view of our results, we used the same statistical approach within limbic system and operculo‐insula regions, using extra‐operculo‐insulo‐limbic structures as the reference, for each category of VES (Table 2).

## Results

Overall, data collected in 203 patients were included (94 women and 109 men, mean age 39.8 years +/− 10.7 years), representing a total of 13,078 stimulations.

### Stimulations

Of the 13,078 stimulations studied, 6,237 (47.7%) were delivered in the temporal lobe, 3,726 (28.5%) in the frontal lobe, 1,177 (9%) in the insula, 948 (7.2%) in the parietal lobe, and 652 (5%) in the occipital lobe. Three hundred and thirty‐eight (2.6%) stimulations could not be precisely localized (due to missing data or MRI) and were excluded from analysis. So, 12,740 stimulations were included in the study; 3,477 (27.3%) were performed using shocks and 9,263 (72.7%) using train stimulations.

None of the 652 stimulations delivered in the occipital lobe evoked VES and stimulations performed in this region were excluded from further analyses.

Finally, 12,088 stimulations were included in statistical analyses, of which 5,752 were performed in the right hemisphere (47.58%) and 6,336 in the left hemisphere (52.41%).

### VES induced by intracerebral stimulations (Table 1)

In total, 543 stimulations evoked VES (4.26% of all stimulations) in 108 distinct patients (53.20%). They were more frequently obtained during right (*n* = 297; 54.7%) than left hemisphere (*n* = 246; 45.3%) stimulations (*p* < 0.0001), and using train stimulations (*p* < 0.0001). Age and gender of patients had no significant influence (*p* = 0.07 and *p* = 0.26 respectively). Stimulations of limbic system and operculo‐insular regions were significantly more associated with VES than those of extra‐operculo‐insulo‐limbic structures (*p* < 0.0001). Brain regions where stimulation was significantly associated with VES (all types of VES taken together) were, for the limbic structures (amygdala (OR = 8.4; [4.5–15.5]), anterior cingulate (OR = 2.7; [1.3–5.9]) and midcingulate cortex (OR = 7.0 [2.3–23.8]), hippocampus (OR = 4.3; [2.3;7.8]), parahippocampus (OR = 3.1; [1.5–6.3]), and temporal pole (OR = 2.5; [1.2–5.0])), and for the operculo‐insular region (anterior (OR = 4.7; [2.6–8.6]) and posterior insula (OR = 6.2; [3.3–12]), frontal operculum (OR = 4.4; [2.2–8.8]), and parietal operculum (OR = 4.3; [2.1–8.9])). This was also true when each type of VES was analyzed individually. The occurrence of viscero‐sensitive, viscero‐vegetative, and emotional responses significantly varied depending on the stimulated lobe and they were also more frequently obtained after stimulation of the limbic system and operculo‐insular regions (*p* < 0.001; *p* < 0.001, and *p* = 0.0006, respectively). Due to the overlap of confidence interval, the hierarchization of significant structures is not possible.

### Distribution of each category of VES within limbic system and operculo‐insular regions (see Table 2)

**Figure 1:**
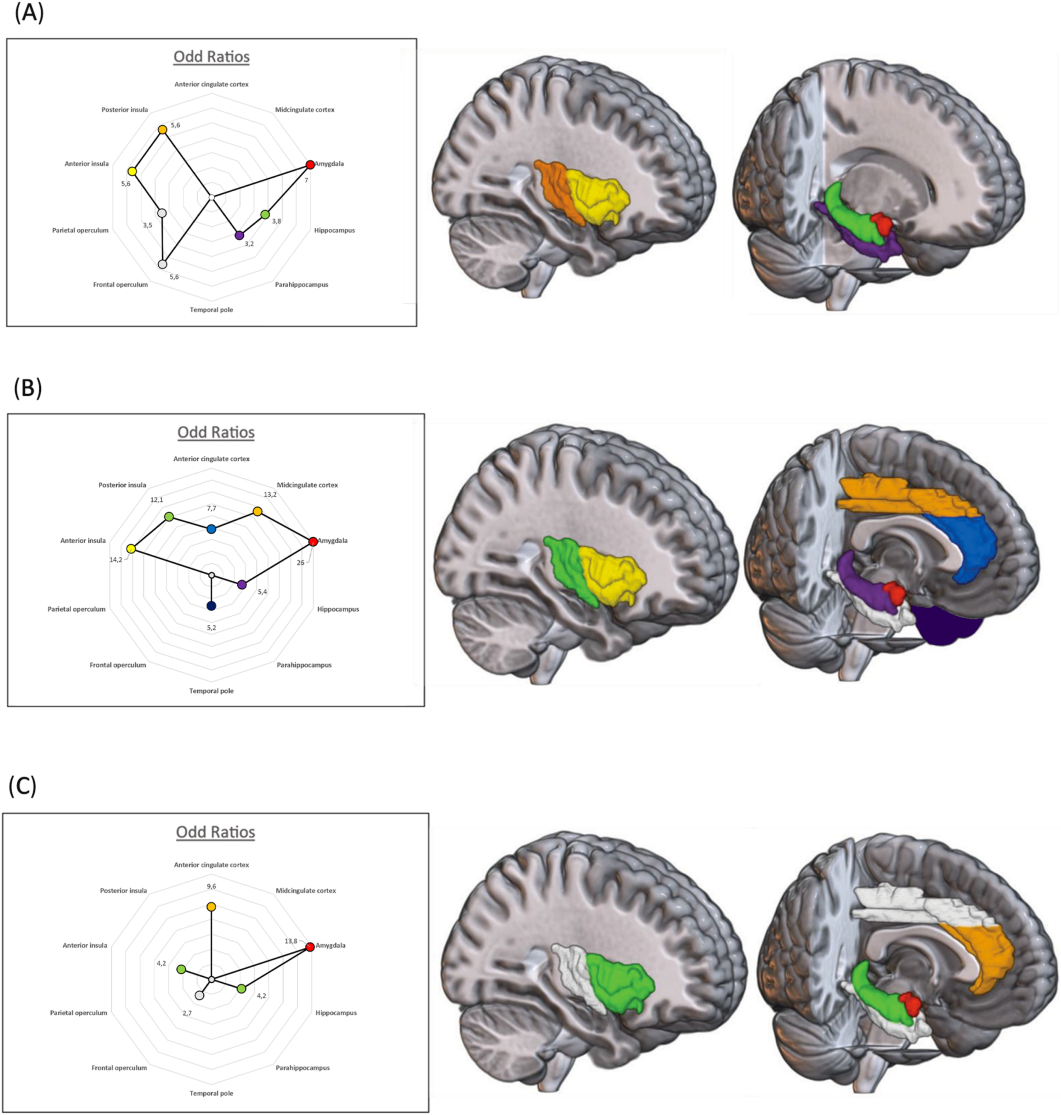
Distribution of the different types of VES in the limbic system and operculo‐insular region: Visceral sensations (A), Viscero‐vegetative sensations (B), and emotional sensations (C). The color of each structure represents its odd ratio (OR), that measures the strength of the statistical relationship between each type of sensation and each stimulated structure.

To illustrate the distribution of each category of VES within limbic system and operculo‐insular regions, we performed a three‐dimensional modeling of this structures with the software MRIcroGl. The ORs of each structure, which measure its statistical relationship with the occurrence of VES, are illustrated in Figure 1. We decided to not illustrate frontal and parietal operculum by 3D modeling for more clarity, as their representation would have overlapped with that of deeply located structures.

#### Viscero‐sensitive sensations

Viscero‐sensitive sensations have been evoked by 325 stimulations (59% of VES) and were more frequently obtained after right hemisphere stimulations (left = 121, right = 204; *p* < 0.001) and using train stimulations (*p* < 0.001). Age and gender had no significant influence (*p* = 0.12 and *p* = 0.50, respectively). They included sensations localized in abdomen, thorax, and throat, such as: abdominal pain (*n* = 31/325; 9.5%), ascending epigastric sensations (*n* = 94/325; 28.9%), abdominal discomfort (*n* = 152/325; 46.7%), throat constrictions (*n* = 68/325; 20.9%), and thoracic sensations (*n* = 92/325; 28.3%).

Within the limbic system, amygdala was the structure where stimulations evoked significantly more frequently viscero‐sensitive sensations with the highest odd ratio (OR = 7; [4.7–12.9]), followed by hippocampus (OR = 3.8; [2.3–6.4]) and parahippocampus (OR = 3.2; [1.5–6.7]). On the contrary, the anterior, the mid‐cingulate cortex, and the temporal pole were not statistically associated with viscero‐sensitive sensations. Within operculo‐insular regions, stimulations of the posterior insula (OR = 5.6; [3.1–10]), anterior insula (OR = 5.6; [3.6–8.7]), frontal operculum (OR = 5.6 [3.2–10]), and the parietal operculum (OR = 3.5 [1.7–7.3]) were associated with viscero‐sensitive sensations (Fig. 1A).

#### Viscero‐vegetative sensations

Viscero‐vegetative sensations were evoked by 103 stimulations (18.9% of VES) without any difference regarding the stimulated hemisphere (left *n* = 54, right *n* = 49; *p* = 0.99), the age (*p* = 0.14), the gender of patients (*p* = 0.37), or the type of stimulation (*p* = 0.11). They included flush (*n* = 52/103; 50.4%), nausea (*n* = 25/103; 24.3%), feeling of tachycardia (*n* = 19/103; 18.4%), and dyspnea (*n* = 12/103; 11.6%).

Amygdala was the structure that most significantly associated with the evocation of viscero‐vegetative sensations, with the highest odd ratio (OR = 26; [11.5–61]). Midcingulate cortex (OR = 13.2; [1.3–95]), anterior cingulate cortex (OR = 7.7; [1.9–31]), hippocampus (OR = 5.4; [2.2–13.2]), and the temporal pole (OR = 5.2; [1.8–15.3]), were also significant. Parahippocampus was not statistically significantly associated with viscero‐vegetative sensations (Fig. 1B).

Within operculo‐insular regions, anterior insula (OR = 14.2; [6.1–33.2]) and posterior insula (OR = 12.1; [4.2–34]) were the unique structure significantly associated with viscero‐vegetative sensations.

#### Emotional sensations

Emotional sensations were obtained after 115 stimulations (21.1% of VES) and were significantly more frequently evoked in women (*p* = 0.029), using train stimulations (*p* < 0.0001) and by stimulating the right hemisphere (left *n* = 50, right *n* = 65; *p* = 0.05). They consisted of a feeling of anxiety (*n* = 91/115; 79.13%), fear (*n* = 23/115; 20%), or sadness (*n* = 1/115; 0.86%). No positive feeling or mirth was elicited.

Three limbic structures were strongly associated with emotional sensations: the amygdala (OR = 13.8; [5.6–33]), the anterior cingulate cortex (OR = 9.6; [4.0–23]), and hippocampus (OR = 4.2 [1.8–9.6]). In operculo‐insular region, anterior insula (OR = 4.2; [2.1–8.3]) and the frontal operculum (OR = 2.7; [1.1–7.7]) were also statistically correlated with emotional responses. Stimulations of the midcingulate cortex and parietal operculum did not evoke any emotional response. The OR was also not statistically significant for parahippocampus, temporal pole and posterior insula. (see Fig. 1C).

Interestingly, 52.1% (*n* = 60) of emotional sensations evoked by stimulations were associated with visceral sensations such as ascending epigastric sensations *n* = 16 (26.6%), throat and thoracic constrictions *n* = 14 (23.3%), abdominal discomfort *n* = 11 (18.3%), abdominal pain *n* = 3 (5%) or flush *n* = 13 (21.6%), and nausea *n* = 2 (3.3%). 47.8% (*n* = 55) consisted of “pure” emotional sensations. There was no difference between emotional sensations associated to visceral sensations and “pure” emotional sensations, in terms of distribution between limbic system, operculo‐insular regions, and extra‐operculo‐insulo‐limbic structures (*p* = 0.059 and *p* = 0.058).

### Focus on throat constriction, ascendant epigastric, and abdominal pain sensations

**Figure 2:**
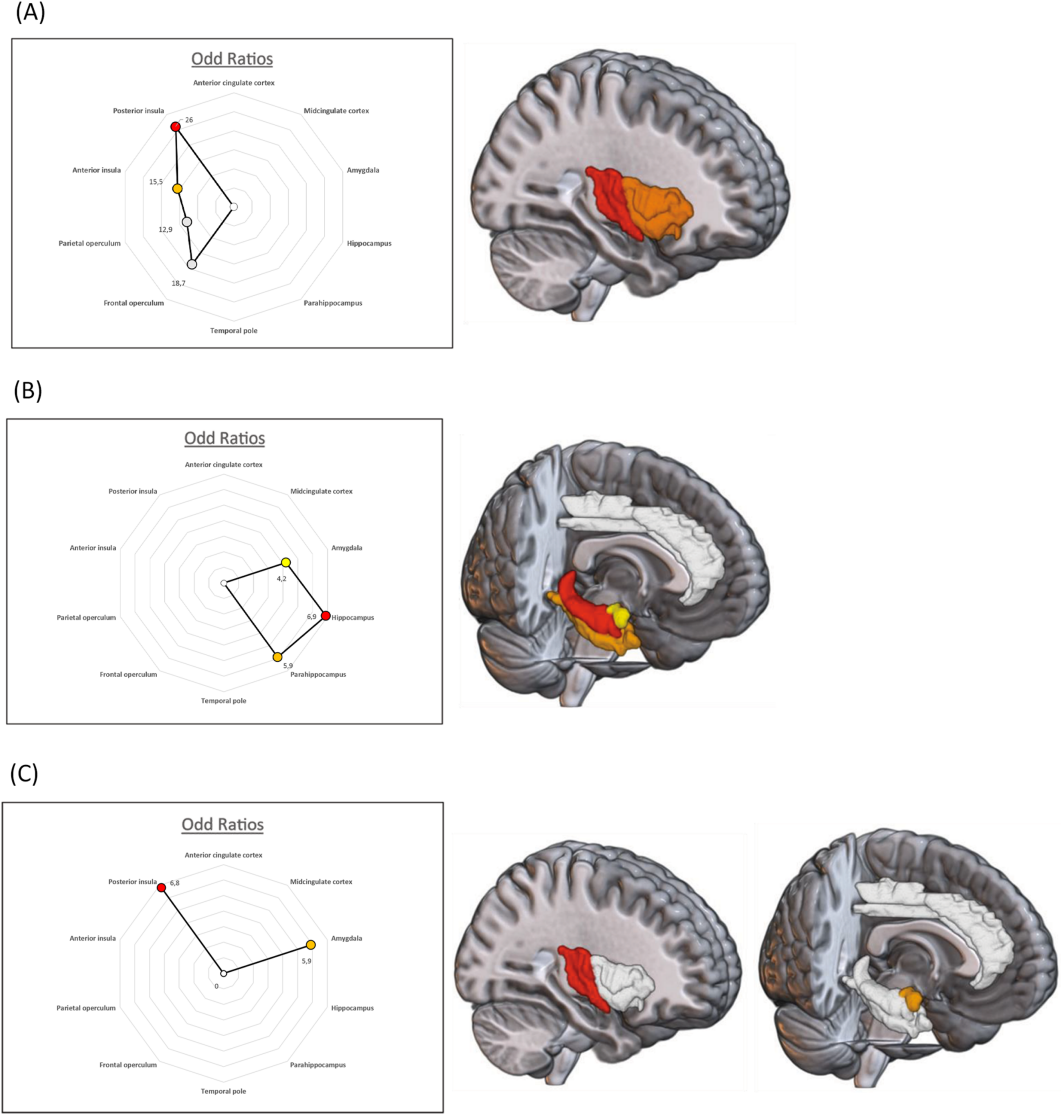
Distribution of throat constrictions (A), epigastric ascendant sensations (B), and abdominal pain (C) in the limbic system and operculo‐insular region. The color of each structure represents its odd ratio (OR), that measures the strength of the statistical relationship between each type of sensation and each stimulated structure.

As other viscero‐sensitive sensations, throat constrictions, ascending epigastric sensations, and abdominal pain were significantly more often obtained after stimulation of limbic structures and operculo‐insular region than after that of extra‐operculo‐insulo‐limbic structures (*p* < 0.0001). As illustrated in Figure 2, these visceral sensations seemed to be relatively specific to the stimulation of some specific structures.

#### Throat and laryngeal constriction

The sensation of laryngeal or throat constriction was elicited 68 times (20.9% of viscero‐sensitive sensations). Within operculo‐insular regions, all structures were associated with throat constriction (anterior insula OR = 15.5 [5.8–41.5], posterior insula, OR = 26 [14.1–63.4], frontal operculum (OR = 18.7 [5.5–63.4]), and parietal operculum (OR = 12.9 [3.1–52.7])) (Fig. 2A). Concerning the limbic system, none of the structures were significantly associated with these type of sensations.

#### Ascending epigastric sensations

Ascending epigastric sensation was evoked by 94 stimulations (28.9% of viscero‐sensitive sensations). Structures that were significantly associated with this sensation were located in the temporo‐mesial region: hippocampus (OR = 6.9 [3.0–15.7]), parahippocampus (OR = 5.9 [2.0–17.0]), and amygdala (OR = 4.2 [1.6–10.8]) (see Fig. 2B).

#### Abdominal pain

Abdominal pain was rarely evoked (31 out of 325 stimulations; 9.5% of viscero‐sensitive sensations). The only two structures associated with abdominal pain were amygdala (OR = 5.9 [1.8–19.0]) and posterior insula (OR = 6.8 [1.4–32.8]) (see Fig. 2C).

## Discussion

To date, the underlying mechanisms of visceral sensations and the location of brain areas involved in their genesis remain unclear. Several studies using functional imaging focusing on the perception of heartbeat or intravesical fullness have shown the implication of many structures such as cingulate cortex, insula, ventromedial prefrontal cortex, and cerebellum.^19^, ^20^ Other studies using rectal or gastric distension suggest the involvement of the insula, the anterior cingulate cortex, the amygdala, the thalamus, the frontal cortex, and the primary and secondary somatosensory cortices in the perception of visceral stimuli.^21^, ^22^, ^23^ However, in most of functional imaging studies, techniques used to activate visceral or viscero‐vegetative processing often also trigger somato‐sensory perception or associate different cognitive or emotional tasks, which make the results difficult to interpret.^20^

We systematically reviewed visceral responses evoked by 13,078 electrical stimulations performed all over the cortex. To our knowledge, this is the first study investigating specifically this question, with such a large sample of stimulations. It appeared clearly that the limbic system and operculo‐insular region were the most involved in VES responses and that some types of visceral responses were preferentially evoked by stimulation of distinct regions of these structures. Amygdala was most strongly associated to all types of VES.

First, in the limbic system, temporo‐mesial regions (hippocampus/parahippocampus) were associated to viscero‐sensitive sensations; anterior, mid‐cingulate cortex, and temporal pole to viscero‐vegetative sensations. The anterior cingulate cortex and hippocampus were associated with emotional sensations.

Then, concerning operculo‐insular region, all of these structures were associated with viscero‐sensitive sensations. Anterior and posterior insula were the only structure associated with viscero‐vegetative sensations. Anterior insula and frontal operculum appeared to be significantly associated with emotional sensations.

### VES and stimulations studies

Since the middle of the 20th century, several studies have reported VES using electrical cortical stimulations.^2^, ^3^, ^13^, ^14^, ^15^, ^16^, ^24^ In accordance with our data, stimulation of mesial temporal regions, in particular, the amygdala, has been shown to induce the three subtypes of VES individualized in our study. Viscero‐sensitive sensations such as abdominal sensations ranging from tenseness, rolling movements, tingling, warmth, to pain could be evoked, mostly referred to the midline of abdomen, with an ascending progression or not.^16^ Vegetative changes were also reported as nausea, flushing, pallor, unusual awareness of heart beats, salivation, or sweating.^16^

Emotional sensations have also been particularly studied^2^, ^16^, ^24^, ^25^ (review in Guillory and Bujarski^26^). Meletti et al^24^ studied emotional responses to stimulation of the temporal lobe. As in our study, they observed mainly fear (which represented 84.4% of the emotional responses), mostly after stimulation of the amygdala and hippocampus, and infrequently after lateral neocortical stimulations. Rarely they reported sadness and happy‐pleasant feelings that we did not obtain, possibly because these feelings were not spontaneously reported by patients. A few studies have also reported laughter and positive emotions elicited by supplementary motor area stimulation^27^, ^28^, ^29^ but, in our study, we did not find any emotional sensations elicited by stimulations in this specific region.

A lateralization of emotional responses to amygdala stimulation has been reported by some authors, with the evocation of negative emotions after right amygdala stimulation, whereas left amygdala stimulation was able to induce either pleasant or unpleasant emotions.^25^ Interestingly, in agreement with our results, these authors observed significantly more frequently emotional responses (and fear in particular) in women than in men, although no gender difference was observed for non‐emotional responses. We also observed this gender difference in fear expression that deserves further investigation to elucidate possible explanations.

Penfield and Faulk^13^ were the first authors who reported a high frequency of visceral responses to stimulation of the antero‐inferior insular quadrant, that they could explore after surgical removal of the temporal operculum. More recent studies reported that VES represent from 5% to 32% of clinical responses to insula stimulation.^3^, ^14^ Stephani et al.,^30^ Pugnaghni et al.,^31^ Krolak‐Salmon et al.,^32^ showed that the insula is an important area for the VES which are very similar to that obtained after stimulation of temporo‐mesial lobe, consisting either of viscero‐sensitive sensations (mainly constrictive sensations located in the pharyngo‐laryngeal, retrosternal or abdominal region), of viscero‐vegetative sensations including salivation, facial blush or dyspnea, or of viscero‐psychic symptoms with feelings of anxiety or fear. Throat constrictions appeared to be associated with insula and frontoparietal operculum stimulations as already reported^33^ and interestingly seemed to be specific to the stimulation of these regions. The VES frequency was not found significantly different between anterior and posterior insular regions in our study; this pertains to the facts that the antero‐inferior quadrant of the insular cortex was underexplored (see below “limitations of the study”) and that we chose the central insular sulcus, around which VES were frequently obtained, as the limit between anterior and posterior insular regions in our segmentation of regions of interest.

We observed a strong correlation between emotional responses and stimulations of the anterior cingulate cortex. Stimulations of the cingulate cortex are known to elicit emotional sensations, mainly after stimulation of the anterior cingulate in its pregenual part, although some responses can also be elicited after mid‐cingulate stimulation. Caruana et al^15^ reported that 7.7% of stimulations of the cingulate cortex, mostly in its ventral sector of pregenual anterior portion, produced fear or anxiety, associated with autonomic changes such as hot flushes in the face, cold sweats, shivers, and tachycardia, while 8% of stimulations in the same region elicited mirth and mirthless laughter. Laughter, smile, and positive emotional sensations have been reported by Caruana et al.,^34^, ^35^, ^36^ Sperli et al.,^37^ Oane et al.^38^ elicited by the stimulation of the anterior cingulate cortex. Bijanki et al.^39^ reported not only smile and laugh but also positive affect and anxiety reduction by the stimulation of the adjacent anterior cingulate bundle. The reason why cingulate stimulations never produced such positive emotions in our study remains unknown.

In 2008, Maliia et al.^33^ showed that the most frequent effects evoked by stimulation of the frontal operculum were language related; the parietal operculum produced mainly somatosensory effects, while the temporal evoked auditory semiology. In our study, the frontoparietal operculum appeared to be also significantly associated with VES and more particularly for visceral sensations (frontal and parietal part) and emotional sensations (frontal part).

Because we analyzed a large number of stimulations delivered to all cortical lobes, our study provides a complete map of regions where stimulations produced visceral or emotional responses and suggest that no other region outside the limbic system and operculo‐insular region are associated with VES. The insula, frontoparieto operculum, and limbic system being strongly connected between each other.^40^

### The localizing value of VES in epilepsy

Direct electrical stimulations of the cortex in the context of epilepsy surgery are used to trigger clinical phenomena reproducing the symptoms of spontaneous seizures, and to obtain an individual functional mapping of the cortex based on clinical responses produced in the absence of induced epileptic activity. The VES responses provides localizing information when such symptoms occur during spontaneous epileptic seizures.

Visceral sensations were frequently reported in focal epilepsies, mostly in mesial temporal,^3^, ^4^, ^5^ insular,^2^, ^4^, ^8^ and frontal epilepsies.^9^, ^10^, ^11^, ^12^ Our stimulation data, confirm that the implication of limbic system and insulo‐opercular region should be considered when a visceral sensation is reported during a focal seizure. The highlighting of specific preferential implication of some brain structures, depending on the type of visceral sensation can help to guide SEEG explorations when visceral or emotional symptoms are part of the ictal semiology. In seizures with viscero‐sensitive aura, an implication of temporo‐mesial structures and of the operculo‐insular region should be preferentially considered. When viscero‐vegetative sensations are reported, a discharge involving amygdala, temporal pole, insula, anterior or mid‐cingulate cortex should be suspected, while emotional sensations evoke mostly the implication of temporo‐mesial structure, anterior cingulate cortex, and frontal operculum. On the basis of our data, we confirm that two ictal symptoms have a high localizing value as suggested by previous analyses of ictal semiology^6^, ^7^: (i) throat or laryngeal constriction was the only VES specific of insulo‐opercular stimulation that showed no correlation with stimulations of other regions of the limbic system including the amygdala (Fig. 2A) and has a high localizing value; and (ii) the ascending epigastric sensation that was clearly associated to stimulation of mesial temporal structures. Paroxysmal abdominal pain is a rare ictal symptom of focal seizures, associated or not to other symptoms such as nausea, vomiting, diarrhea.^41^ To date, the localizing value of this symptom remains uncertain, a few scalp of invasive EEG recordings have suggested various origins including temporal, parietal or frontal lobes, and insulo‐opercular cortex.^42^, ^43^, ^44^ The relationship we found between abdominal pain responses and stimulation of amygdala and insula suggests a predominant role of these two regions in the genesis of ictal abdominal pain.

### Visceroception, interoception, and emotions

The visceral sensations that we collected in response to electrical stimulation, in particular viscero‐sensitive and vegetative sensations, refer to the concept of visceroception as part of interoception defined as the sense of the internal state of the body.^45^, ^46^ Contemporary definition of interoception encompasses visceroception but more broadly relates to all physiological tissues that relay to the central nervous system signals providing information on the physiological state of the body, including cardiovascular, gastrointestinal, genitourinary, nociceptive, somato‐sensory, thermoregulatory, endocrine, and immune systems.^45^, ^46^

Interoception is thus crucial for maintaining homeostasis conditions in the body and, potentially provides bodily symptoms which are critical for human emotion and self‐awareness.^45^, ^47^ William James^48^ was the first author to propose a psychological theory linking these bodily symptoms to emotional experience and the perception of signals arising from the body plays not only an important, but not an exclusive role in many theories of emotions.^45^, ^48^, ^49^ Interestingly, the neural network of cortical areas, grouped as limbic system and operculo‐insular regions, where focal stimulations produced viscero‐sensitive and viscero‐vegetative sensations is largely co‐extensive with that of areas involved in the genesis of emotions.^50^ In spite of this co‐localization in the brain of emotional and visceral representations, stimulations reported in this study rarely produced an association of emotional and visceral sensations (60/543, 11%) and “pure” emotional responses without any visceral sensations were almost as frequent (55/543, 10.1%). This probably pertains to the fact that very focal stimulations do not reproduce responses resulting from integrated activation of the cortical circuitry involved in the genesis of emotions and can dissociate bodily symptoms and emotional feelings. Such a dissociation was observed for instance in the anterior cingulate cortex where a correlation was found with emotional but not with visceral responses to stimulation. Another discrepancy between the “visceral brain” as assessed by cortical stimulations and the “emotional brain” mapped by neuroimaging is that stimulations of the mesial prefrontal and orbito‐frontal cortex did not correlate with visceral and emotional responses, while this brain region is part of the emotional cortical network.^39^ One reason might be that, this region is not involved in reception of interoceptive inputs and plays a specific role to engage an appropriate behavior in a given emotional context.

### Limitations of the study

Electrodes implantations aimed at delineating the epileptogenic zone in the context of epilepsy surgery, so that only part of the cerebral cortex was explored in each individual patient. Therefore. exhaustive study of the cortex was made possible by pooling stimulation data from a large population of patients. Using mostly electrodes implanted orthogonal to the mid‐sagittal plane, the antero‐inferior portion of the insula was less frequently explored than other insular regions because of the emergence of sylvian vessels, so that we may have underestimated the frequency of visceral responses in the antero‐inferior insular cortex.

Another limitation of this retrospective study is that no systematic questionnaire on VES has been carried out during stimulations. Therefore, the frequency of VES responses might have been underestimated because patients may have not spontaneously reported light VES occurring at the same time as other more “salient” symptoms. Likely, some of the clinical characteristics of VES were not always precisely detailed, such as the exact localization of abdominal sensation or pain.

## Conclusion

Most of regions where stimulation produced visceral and emotional sensations belong to the limbic system or operculo‐insular regions, among which amygdala is the structure that was most strongly linked with the production of all types of visceral and emotional responses. The others structures were preferentially, but not exclusively, linked with more specific types of responses: temporo‐mesial structures and operculo‐insular regions for viscero‐sensitive sensations; insula, anterior cingulate cortex, mid‐cingulate cortex, and temporal pole for viscero‐vegetative sensations; temporo‐mesial structures, anterior cingulate cortex, and frontal operculum for emotional sensations. These findings can help to guide SEEG explorations when visceral or emotional symptoms are part of the ictal semiology. Our data support some co‐localization of visceral and emotional representations in the human brain. However, if most of our visceral sensations are coupled with our emotional feelings, most of focal electrical stimulations dissociate visceroception from emotional experience.

## Author Contributions

H.S., L.M., and F.M. conceptualized and designed the study. H.S., L.M., F.M., S.R., A.M., H.C, J.I, M.G., and M.H. acquired and analyzed the data. H.S., L.M., F.M., and S.R. drafted the text and prepared figures.

## Conflicts of Interest

The authors have no conflict of interest to declare.
